# PSTK exerts protective role in cisplatin‐tubular cell injury via BAX/BCL2/Caspase3 pathway

**DOI:** 10.14814/phy2.70162

**Published:** 2025-01-10

**Authors:** Yifan Wu, Yuanyuan Xv, Limei Zhao, Ziqi Zhou, Miaomiao Wang, Jima Xi, Ying Liming, Jianling Gao, Bingqing Deng, Dong Zheng

**Affiliations:** ^1^ Department of Pathology and Pathophysiology Medical College of Soochow University Suzhou Jiangsu China; ^2^ Department of Thoracic Surgery, School of Medicine Shanghai Chest Hospital, Shanghai Jiao Tong University Shanghai China; ^3^ School of Biology & Basic Medical Sciences, Medical College of Soochow University Suzhou Jiangsu China; ^4^ Department of Oncology Eastern Hepatobiliary Surgery Hospital Shanghai China; ^5^ Liangxi Taihu Hospital of Traditional Chinese Medicine Wuxi Jiangsu China; ^6^ National Heart and Lung Institute Imperial College London London UK; ^7^ Department of Intensive Care Suzhou 4th People's Hospital, Soochow University Suzhou China; ^8^ The Department of Nephrology Suzhou 4th People's Hospital, Soochow University Suzhou China

**Keywords:** AKI, apoptosis, cisplatin, oxidative stress, PSTK

## Abstract

Cisplatin is a widely used anticancer drug, but its accumulation in renal tubular epithelial cells (TECs) can cause acute kidney injury. Phosphoseryl‐tRNA kinase (PSTK) is an intermediate product produced under oxidative stress conditions. This study aimed to elucidate whether PSTK could protect TECs and its possible mechanisms. We found that PSTK levels decreased after cisplatin treatment, but PSTK overexpression using lentivirus vectors protected TEC viability. Overexpression of PSTK increased selenoprotein concentrations and reduced intracellular ROS levels. Additionally, PSTK overexpression inhibited the BAX/BCL2/Caspase 3 pathway after cisplatin stimulation, suggesting its potential role in preventing cell apoptosis. Taken together, this study suggests that PSTK could protect TEC viability from cisplatin‐induced injury, possibly by inhibiting mitochondrial apoptosis. The study is significant for developing therapeutic strategies that could manipulate PSTK to delay AKI progression.

## 1 INTRODUCTION

Acute kidney injury (AKI), a clinical syndrome characterized by an abrupt loss of kidney function within hours, is a global health problem with high mortality (Turgut et al., 2023). Despite significant tremendous efforts by nephrologists over the past decades, therapeutic strategies for AKI patients remain limited (Liu et al., 2021). Therefore, understanding underlying mechanisms of AKI is essential for developing effective prevention and treatment of strategies. The pathogenic factors for AKI primarily include ischemia (Turgut et al., 2023), renal toxic reagents (Perazella & Rosner, 2022), and sepsis (Singer et al., 2016). Several causes, such as tubular cell injury, peritubular endothelial dysfunction, and inflammatory cell infiltration, contribute to the initiation and progression of AKI (Guzzi et al., 2019), with tubular cell injury being considered to be the most pivotal one (Liu et al., 2018). Additionally, oxidative stress plays a critical role tubular cell injury during AKI. The high energy demand of tubular cells under physiological condition, supported by mitochondria under physiological conditions, can lead to mitochondrial dysfunction, contributing tubular cell injury and death (Brooks et al., 2009; Parikh et al., 2015).

Phosphoseryl‐tRNA kinase (PSTK) is an intermediate enzyme that changes rapidly under oxidative stress conditions. PSTK's is to participate in the synthesis of antioxidant molecules, such as glutathione peroxidase (GSH‐Px) (Chen et al., 2022) and C‐reactive protein (CRP) (Sherrer et al., 2011). Selenoproteins were reported to protect kidney injury by alleviating oxidative stress and activating NRF2 transcription factor (Tian et al., 2024). As a phosphoseryl transport ribokinase, PSTK specifically phosphorylates seryl transfer RNA [seryl‐tRNA(Sec)] to O‐phosphorylated serine transfer RNA, forming selenocysteine intermediate transport RNA (Maenpaa & Bernfield, 1970). However, the specific functional mechanisms of PSTK in animal and human diseases are not fully understood. We previously reported that PSTK participates in mitochondrial complex I assembly, mitochondrial apoptosis, and mitochondrial fatty acid β‐oxidation in oxidative tissue injury (Zheng et al., 2015).

In this study, we aimed to determine whether PSTK could protect tubular epithelial cells (TECs) from cisplatin‐induced renal injury. Our results showed that PSTK could protect TEC proliferation through the BAX/BCL2/CAS3 pathway, a classical mitochondrial apoptosis pathway.

## 2 RESULTS

### 2.1 Cisplatin decreased PSTK in TECs

**FIGURE 1:**
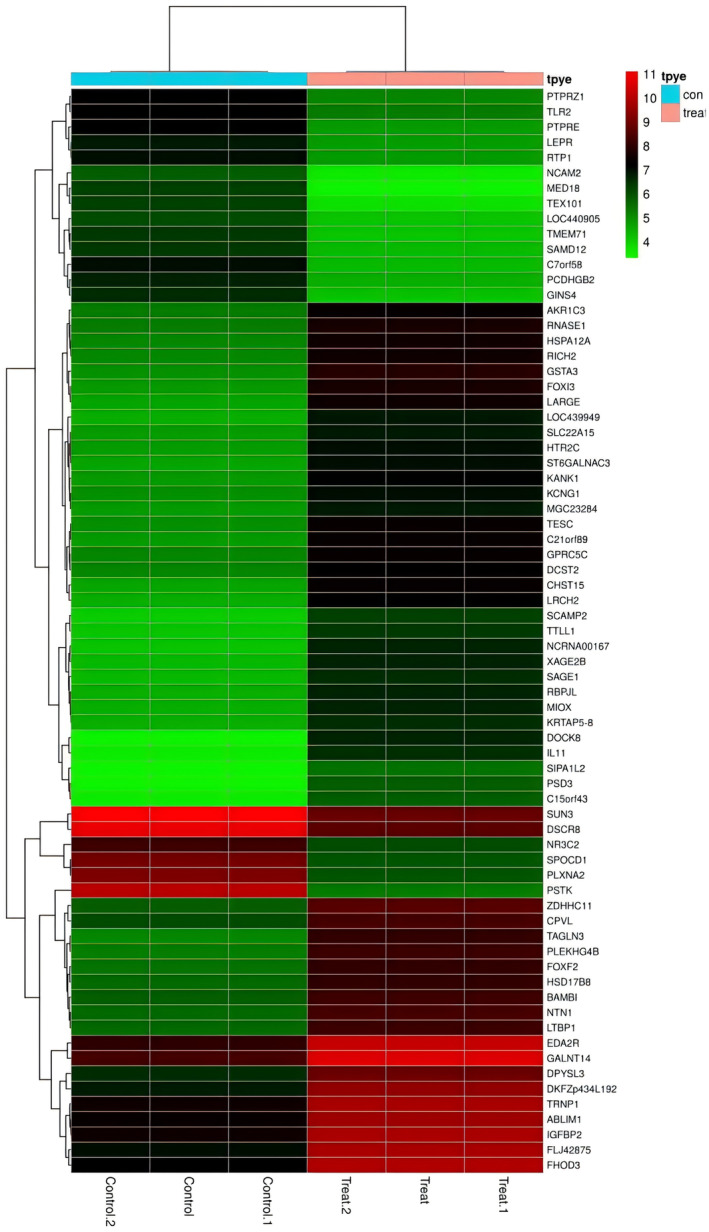
Differential genes involved in cisplatin induced TECs injury.

**FIGURE 2:**
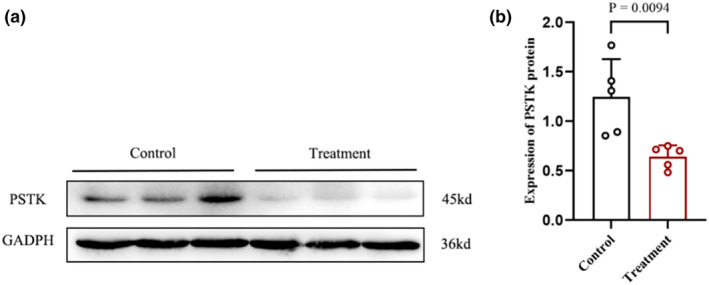
Cisplatin inhibited PSTK expression in TECs. (a, b) Western blot analysis and quantitative assessment of PSTK protein levels in normal and cisplatin‐treated TECs (*n* = 5). All the data were repeated three times and represented as the mean ± SEM, *p*‐value was determined by one‐way ANOVA with Dunnett's post hoc correction.

To examine differential genes involved in cisplatin‐induced TEC injury, we used throughout mRNA sequencing to identify the significantly altered genes. Among these, PSTK was the only gene implicated in the synthesis of selenocysteine, an essential trace element and micronutrient found primarily in selenoproteins (Figure 1). We have performed a GO analysis of proteomics of overexpression of PSTK, which showed that mitochondrial function is closely correlated with the levels of PSTK. Furthermore, we conducted a phosphotomic study on PSTK overexpression, and the data explained that after PSTK overexpression, the levels of phosphorylation related to oxidative stress and mitochondrial function are highly associated with those of PSTK (Supplementary Figure S1). We validated that the protein level of PSTK decreased in cisplatin‐treated TECs (Figure 2), suggesting that PSTK could be a key player in the pathogenesis of cisplatin‐induced TEC injury.

### 2.2 PSTK protects TECs after cisplatin treatment

**FIGURE 3:**
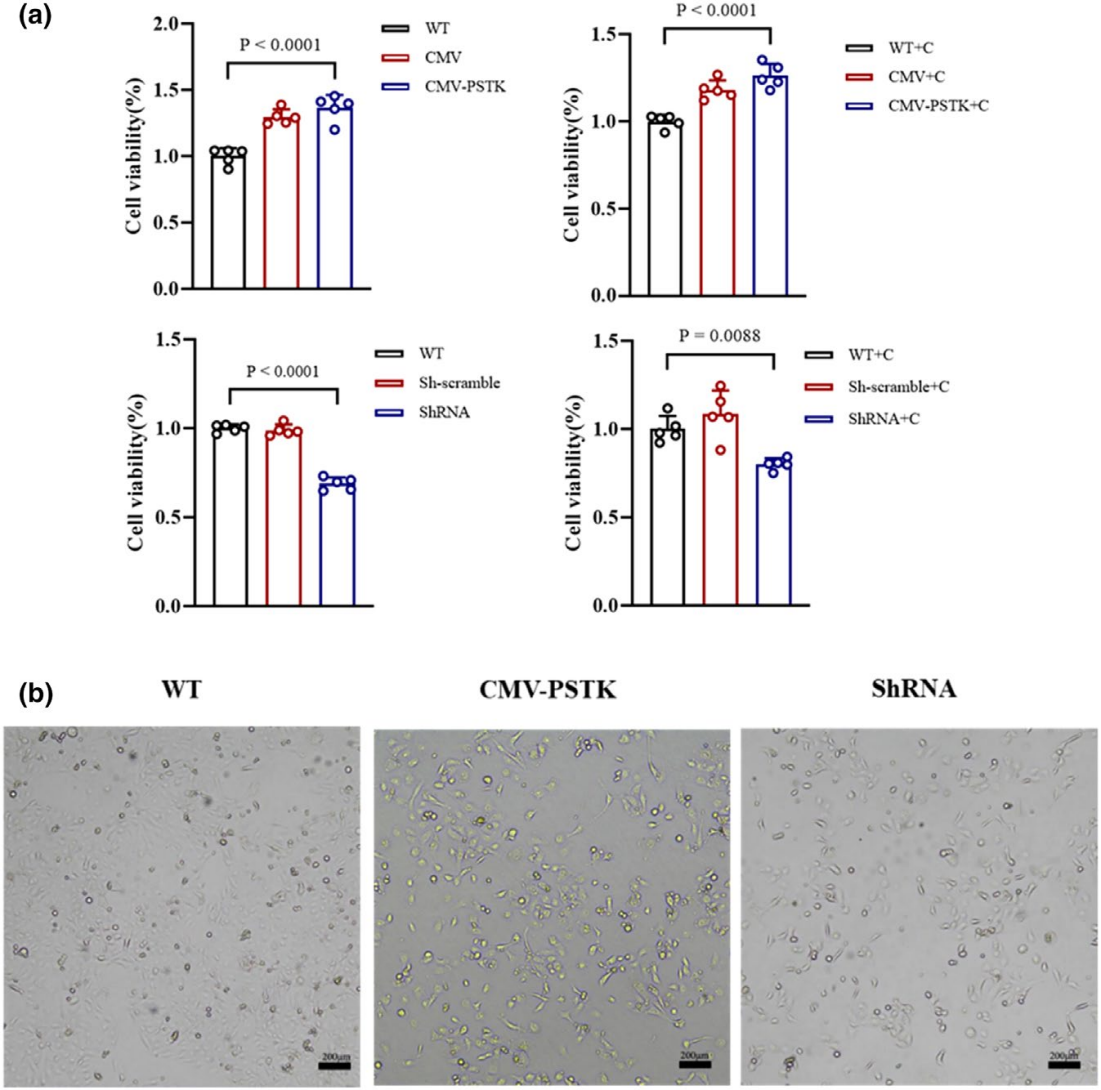
PSTK protected the viability of HK‐2 cells. (a) MTT assay to assess cell viability (*n* = 5, one‐way ANOVA). All the data were repeated 3 times and represented as the mean ± SEM, *p*‐value was determined by one‐way ANOVA with Dunnett's post hoc correction. (b) Bright‐field images of HK‐2 cells containing WT, CMV‐PSTK, ShRNA groups (scale bar = 200 μm).

To further understand the function of PSTK, we used the MTT assay to assess its role on cell viability. PSTK increased cell activity both before and after cisplatin stimulation (Figure 3a). Additionally, the bright‐field microscopy images showed that PSTK promoted cell proliferation to a certain extent, whereas knockdown with PSTK yielded contrary results (Figure 3b). These findings suggest that PSTK overexpression could prevent cisplatin‐induced TEC injury by protecting TEC proliferation, while knockdown of PSTK could exacerbate TEC injury following cisplatin treatment.

### 2.3 PSTK can increase selenoproteins concentration and reduce ROS

**FIGURE 4:**
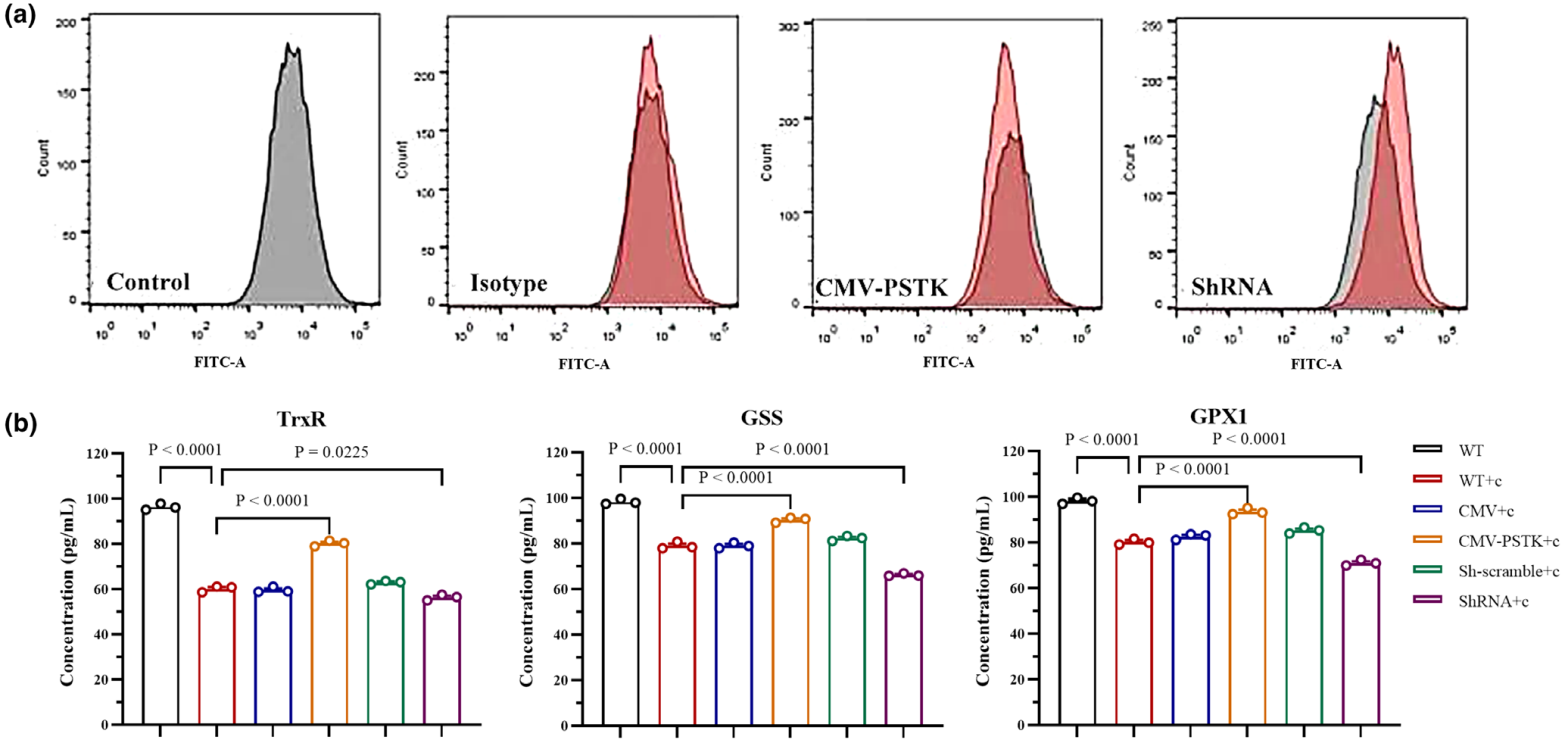
PSTK could reduce intracellular ROS levels. (a) The ROS level in renal cells were detected by flow cytometry (Gray is control group, red is experimental group). The gating strategy of cell population is as follows: Live cells are screened according to forward scattered light (FSC) and lateral scattered light (SSC); Positive populations were then distinguished using DCFH‐DA fluorescence intensity (X‐axis) with appropriate background fluorescence (Y‐axis). A negative population consists of low fluorescence events, and a positive population is defined as a fluorescence intensity greater than a predetermined threshold (in the range of 10^3^–10^5^). (b) The concentration of selenium protein in renal cells were detected by ELISA (*n* = 3). The wild‐type treatment group (WT + c) was compared with wild‐type (WT); The overexpression (CMV‐PSTK) and knockdown treatment (ShRNA+c) groups were compared with the wild‐type treatment group (WT + c). All the data were repeated three times and represented as the mean ± SEM, *p*‐value was determined by one‐way ANOVA with Dunnett's post hoc correction.

To evaluate the effect of PSTK on intracellular selenoproteins concentration and ROS levels, we used ELISA and flow cytometry to measure cellular levels of selenoproteins Thioredoxin Reductase (TrxR), Glutathione Synthetase (GSS), Glutathione Peroxidase 1 (GPX1), and reactive oxygen species. Following cisplatin treatment, the concentrations of TrxR, GSS, and GPX1 all declined. However, selenoprotein concentrations increased in the PSTK overexpression group but decreased in the knockdown group compared to the cisplatin control group (Figure 4a). Meanwhile, ROS levels were elevated in cisplatin treated TECs. Overexpression of PSTK reduced intracellular ROS levels, whereas PSTK knockdown exacerbated ROS levels (Figure 4b). In addition, mitochondrial ROS levels were detected in WT, CMV and CMV‐PSTK cells before and after cisplatin treatment, and the results showed that overexpression of PSTK could effectively reduce mitochondrial ROS production (Supplementary Figure S2). These results suggest PSTK can mitigate intracellular ROS levels by increasing selenoprotein production.

### 2.4 PSTK suppresses BAX/BCL2/CAS3 pathway

**FIGURE 5:**
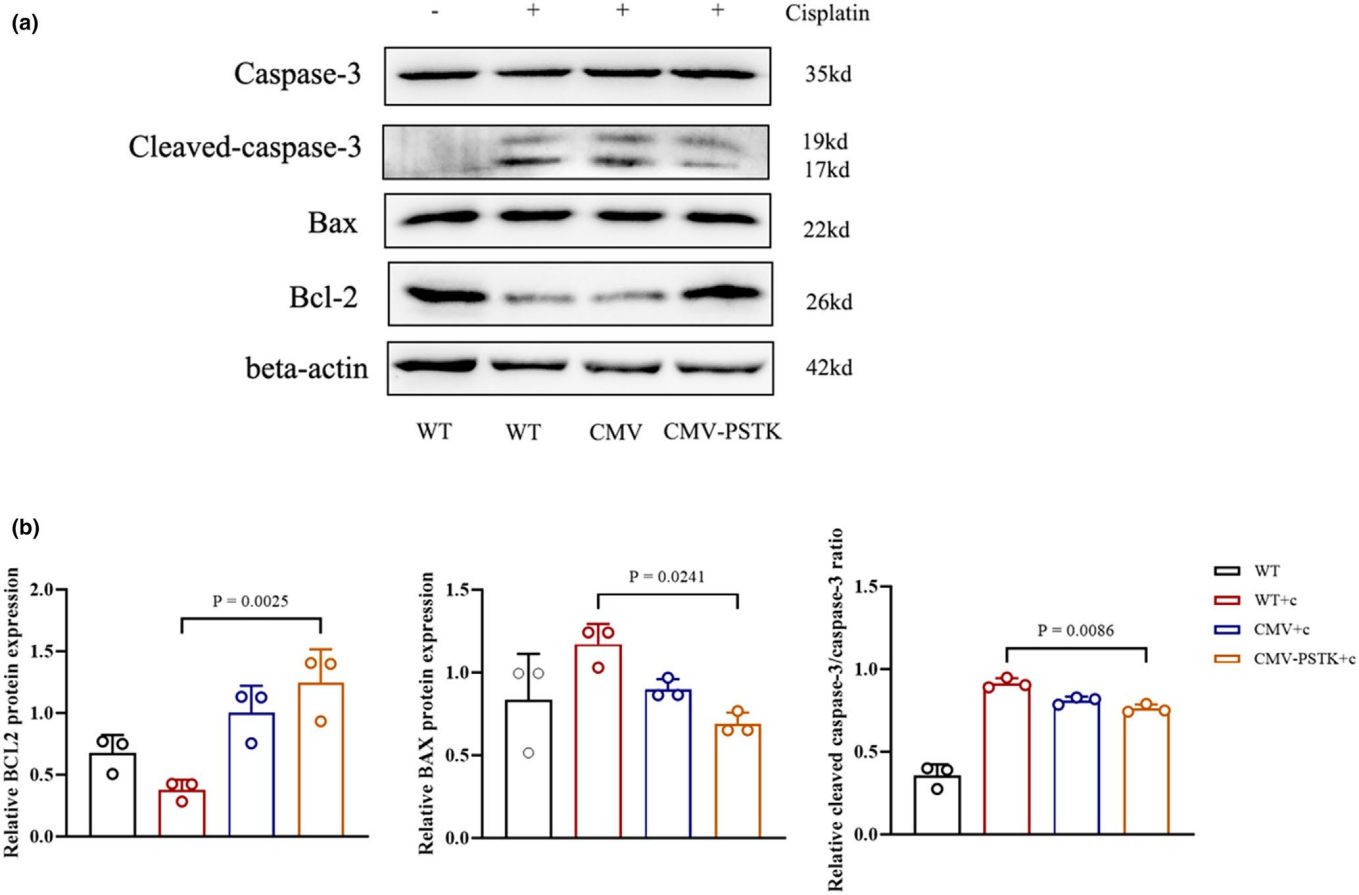
PSTK increased BCL‐2, decreased BAX and cleaved caspase‐3 expression. (a, b) Western blot analysis and quantitative assessment of BAX/BCL2/CAS3 protein in different groups (*n* = 3). All the data were repeated 3 times and represented as the mean ± SEM, *p*‐value was determined by one‐way ANOVA with Dunnett's post hoc correction.

To explore the mechanism by which PSTK suppresses TEC injury, we investigated its effect on the BAX/BCL2/CAS3 pathway, a classical signaling pathway involved in mitochondrial apoptosis triggered ROS. We found that PSTK increased BCL‐2 and decreased BAX and cleaved caspase‐3 levels (Figure 5), indicating that PSTK might prevent mitochondrial apoptosis in tubular cells.

## 3 DISCUSSION

In this study we found that cisplatin decreased PSTK levels in renal tubular epithelial cells. PSTK was capable of protecting TECs, by increasing selenoprotein levels and reducing ROS. Most importantly, PSTK inhibited the BAX/BCL2/CAS3 pathway, which plays a crucial role in mitochondrial apoptosis.

Cisplatin‐induced TEC injury is an ideal model for studying AKI (Ozkok & Edelstein, 2014). This study is the first to demonstrate that PSTK has a significant protective role in this model. While extensive research has identified various treatments for the treatment of AKI (Pabla & Dong, 2008). The 21st amino acid, selenocysteine, biosynthesized by PSTK through phosphorylation of the hydroxyl group of the selenocysteine‐specific tRNA (Bock et al., 1991; Carlson et al., 2004), has a lower reduction potential than cysteine, making PSTK a good candidate for antioxidant activity. Therefore, PSTK could be a potential target for AKI treatment.

Oxidative stress, an imbalance between the production of reactive oxygen species (ROS) and antioxidant defense (Sies & Cadenas, 1985), is a hallmark of many human diseases (Valko et al., 2007). Therefore, elucidating how this balance is maintained or disrupted in molecular detail. Cisplatin is commonly used to model ROS models both in vitro and in vivo (Pabla & Dong, 2008). Previous studies showed that SelT, one of the selenoproteins, was significantly reduced in cisplatin‐induced AKI, and SelT could silence cisplatin‐induced ROS, proving that SelT protects against cisplatin‐induced AKI by inhibiting oxidative stress and apoptosis (Huang et al., 2020). PSTK participates in the synthesis of selenocysteine—a well‐known antioxidant and key components of selenoprotein (Carlson et al., 2004). In this study, we demonstrated that PSTK could increase selenoprotein levels to counteract ROS‐induced cellular injury. Most importantly, we found that PSTK inhibited the BAX/BCL2/CAS3 pathway by increasing BCL2 levels and decreasing BAX and cleaved caspase‐3. BAX/BCL2/CAS3 pathway is known to be a crucial mechanism for countering mitochondrial ROS (Peng et al., 2021). These findings suggest that PSTK might inhibit mitochondrial apoptosis in renal proximal tubular cells.

In conclusion, we have shown that PSTK could protect TECs, increase selenoprotein levels, and reduce ROS. Most significantly, PSTK could inhibit the BCL2/BAX/CAS3 pathway. Our findings not only provide new insights into the mechanism of ROS protection in AKI but also open new avenues for the developing treatments for oxidative stress in related diseases, such as cancer and Parkinson's disease.

## 4 METHODS

### 4.1 Construction of PSTK overexpression and ShRNA lentiviral vectors

The human PSTK plasmid (gene ID: 118672; NCBI accession: NM_001363531.2) was ligated into the LV‐EFS > hPSTK‐CMV > EGFP/T2A/Puro vector. The shRNA targeting human PSTK (shRNA #1:TTCTTGATGGCTGTCATTAATCTCGAGATTAATGACAGCCATCAAGAA; shRNA #2:CTTCTAGCAGAAGAACTTAACCTCGAGGTTAAGTTCTTCTGCTAGAAG) was ligated into the LV‐U6 > hPSTK [shRNA#1]‐PGK > EGFP/T2A/Puro and LV‐U6 > hPSTK [shRNA#2]‐PGK > EGFP/T2A/Puro vectors. Lentivirus packaging was performed by co‐transfecting the recombinant overexpression plasmid with helper plasmids. The lentivirus vector carrying the empty vector served as a negative control. The DNA mixed with Lipofectamine 2000 (Invitrogen; Thermo Fisher Scientific, Inc.) for 20 min at room temperature and then transfected into the 293 T cells. Subsequently, the lentivirus was harvested.

### 4.2 Culture of TECs and transfection

HK‐2 cells (from Cell Bank, Chinese Academy of Sciences; SCSP‐511) were cultured in DMEM/F12 medium (DMEM, Boster, Wuhan, China) supplemented with 10% fetal bovine serum (FBS, BI, Israel) until they reached logarithmic phase. Cells were then digested with 0.05% trypsin and plated in a six‐well plate (2 × 10^5^ cells/well). In the presence of 4 μg/mL polybrene (C0351, Beyotime, China), the overexpression and knockdown lentivirus particles were used to infect HK‐2 cells for 24 h in serum‐free medium. After 48 h of transfection, cells were selected with adding puromycin (ST551, Beyotime, China) for subsequent analyses. Correct insertion of the synthesized cDNA and shRNA was confirmed by direct sequencing.

### 4.3 Transcriptome sequencing on the Illumina Hiseq. 4000 platform

mRNAs were enriched using oligo(dT)‐coated magnetic beads (S1419S, Biolab New England, Ipswich) and fragmented with fragmentation buffer. Double‐stranded cDNAs were synthesized using random primers (N8080127, Thermofisher Scientific, Epsom Surrey), and the fragments were subsequently attached with sequencing adapters. Transcriptome sequencing was performed on an Illumina Hiseq. 4000 system (San Diego, CA).

### 4.4 Intracellular ROS detected by flow cytometry

Cells were cultured in 6‐well plates and categorized into CMV‐PSTK, shRNA, isotype, and control groups. After incubating with 10 μM 2′,7′‐Dichlorodihydrofluorescein diacetate (DCFH‐DA) fluorescent probes (S0033, Beyotime, China) at 37°C for 30 min, ROS levels were measured using a FACS‐Aria II flow cytometer (BD Biosciences). Excitation wavelength of 488 nm and emission wavelength of 525 nm were used. The gating strategy of cell population is as follows: All cells were first gated with forward‐scattered light (FSC) and side‐scattered light (SSC) to remove debris and aggregates. DCFH‐DA fluorescence intensity was then used to distinguish between positive and negative cell populations. A positive group is defined as having a fluorescence intensity greater than 10^3^ (a 10‐pair value of fluorescence intensity, log scale), while a negative group is defined as having a fluorescence intensity below this threshold. All streaming data were analyzed using FlowJo software.

### 4.5 Elisa

Cells from different groups were collected after treatment, lysed in lysis buffer, and then centrifuged at 3000 rpm for 10 min to obtain the supernatant. The concentration of TrxR, GSS, and GPX1 were measured by using specific human TrxR, GSS, and GPX1 ELISA kits (Fangcheng Biotechnology, Beijing, China) according to the manufacturer's instructions.

### 4.6 RNA extraction and qRT‐PCR

Total RNA was extracted according to the RNA extraction kit (Tiangen Biotech, China) and reverse‐transcribed into cDNA using All‐in‐One First‐Strand Synthesis MasterMix (BestEnzymes, China). Quantitative real‐time PCR was performed with the SYBR qPCR SuperMix Plus kit (Novoprotein, China) on Gentier 96E/96R (TIANLONG). The relative expression of GADPH and PSTK was calculated using $2^{-∆∆C_{t}}$ method. The sequence of primers is as follows: GADPH (forward: 5′‐GGTGTGAACCATGAGAAGTATGA‐3′; reverse: 5′‐GAGTCCTTCCACGATACCAAAG‐3′); PSTK (forward: 5′‐GCCCAGTCTTGCTACCTCTT‐3′; reverse: 5′‐TCAGGTGGATGGTCTCAGGA‐3′).

### 4.7 Western blotting

Western blotting was performed using anti‐PSTK antibody (Santa Cruz, sc‐373,991), anti‐BAX antibody (Abcam, ab32503), anti‐BCL2 (Abcam, ab182858), and anti‐Caspase‐3 (Abcam, ab32351). Total cell lysates were prepared by removing the growth medium and adding RIPA lysis buffer (Solarbio, China), followed by mixing on a rotary mixer for 30 min at 4°C. Cellular debris was pelleted by centrifugation at 10,000 ***g*** for 10 min at 4°C. The supernatant was transferred to a fresh 1.5 mL conical tube on ice. After boiling for 5 min, samples were electrophoresed on a 10% acrylamide gel and transferred onto Hybond P–polyvinylidene fluoride membrane (PVDF, GE Healthcare). The membrane was then blocked with tris‐buffered saline–Tween [Tris 50 mM (pH 8), NaCl 150 mM, 0.1% Tween] supplemented with 5% milk, followed by incubating with primary antibodies for 2 h at 25°C or overnight at 4°C. Membranes were washed and incubated with horseradish peroxidase–conjugated goat anti‐rabbit (Abcam, ab6721) or anti‐mouse antibodies (Santa Cruz, sc‐516,102). Protein bands were imaged by a chemiluminescence kit (Tanon, China) and WB imaging system (SH‐Focus523, China). Relative expression levels were calculated by comparing the intensity of the target protein band with that of an internal reference proteins, such as beta‐actin (Abcam, ab8227) GADPH (Abcam, ab181602).

### 4.8 Cell viability

To examine the protective effects of PSTK against cisplatin‐induced injury in TECs, cells were seeded in 96‐well plates and treated with 15 μg/mL cisplatin for 24 h. After treatment, 10 μL of MTT solution (C0009S, Beyotime, China) (final concentration of 0.5 mg/mL) was added to each well, and the plates were incubated at 37°C in 5% CO_2_ for 4 h. Following incubation, 100 μL methanol solubilization solution (C0009S, Beyotime, China) was added to each well. The plate was incubated at 37°C to ensure complete dissolution. Absorbance was measured at 570 nm using a microplate reader. Wells containing medium, MTT solution, and methanol solubilization solution served as controls. Cell viability was calculated by (OD570 nm of experimental group/OD570 nm of control group) × 100%.

### 4.9 Statistical analysis

Quantitative data are presented as mean ± SEM. Graphpad 7 (La Jolla, CA) was used for statistical analysis. Shapiro–Wilk test was used to test the normality of data distribution, and the data were analyzed by one‐way analysis of variance (ANOVA) for multiple comparisons of parametric data. A multiple‐comparison Dunnett test was carried out when ANOVA differences reached significant results (*p* < 0.05).

## AUTHOR CONTRIBUTIONS

Z.D. designed the research. Z.D., W.Y.F., X.Y.Y., Z.L.M., and Z.Z.Q. performed the research and wrote the paper. W.M.M. and X.J.M. analyzed the data. L.M.Y., G.J.L., and D.B.Q. reviewed and revised the paper. All authors participated in the decision to submit for publication. All authors read and approved the final manuscript.

## CONFLICT OF INTEREST STATEMENT

The authors declare that there is no conflict of interest regarding the publication of this article.

## ETHICS STATEMENT

No ethical concerns are addressed in this paper, as we did not study any human or animal subjects, nor did we collect any personal information or sensitive data.

## Supporting information


Appendix S1.

## Data Availability

All relevant data are within the manuscript and its Additional files.
