# Correction to “PSTK exerts protective role in cisplatin‐tubular cell injury via BAX/BCL2/caspase3 pathway”

**DOI:** 10.14814/phy2.70585

**Published:** 2025-09-30

Wu, Y., Xv, Y., Zhao, L., Zhou, Z., Wang, M., Xi, J., Liming, Y., Gao, J., Deng, B., & Zheng, D. (2025). PSTK exerts protective role incisplatin‐tubular cell injury via BAX/BCL2/Caspase3pathway. *Physiological Reports*, 13, e70162

The authors regret that certain errors in affiliation addresses were not corrected during article proofing. The author's affiliation “^7^Department of Intensive Care, Suzhou 4th People's Hospital, Soochow University, Suzhou, China; ^8^The Department of Nephrology, Suzhou 4th People's Hospital, Soochow University, Suzhou, China;” was incorrect. This should have read “^7^The Department of Critical Care Medicine, The Fourth Affiliated Hospital of Soochow University; ^8^The Department of Nephrology, The Fourth Affiliated Hospital of Soochow University”

The affiliation for correspondence “Bingqing Deng, The Department of Nephrology, Suzhou 4th People's Hospital, Soochow University, Suzhou, China. Email: deng.bingqing@tongji.edu.cn” was incorrect.

This should have read “Bingqing Deng, The Department of Nephrology, The Fourth Affiliated Hospital of Soochow University, Suzhou, China. Email: deng.bingqing@tongji.edu.cn”

The authors apologize for these errors.

