# Supplementary material for: PSTK exerts protective role in cisplatin‐tubular cell injury via BAX/BCL2/Caspase3 pathway
Source: Physiol Rep. 2025 Jan 10;13(1):e70162. doi: 10.14814/phy2.70162 (PMC11723822; doi:10.14814/phy2.70162)
Supplement: Supplementary file 1 — Appendix S1. [file PHY2-13-e70162-s001.docx]

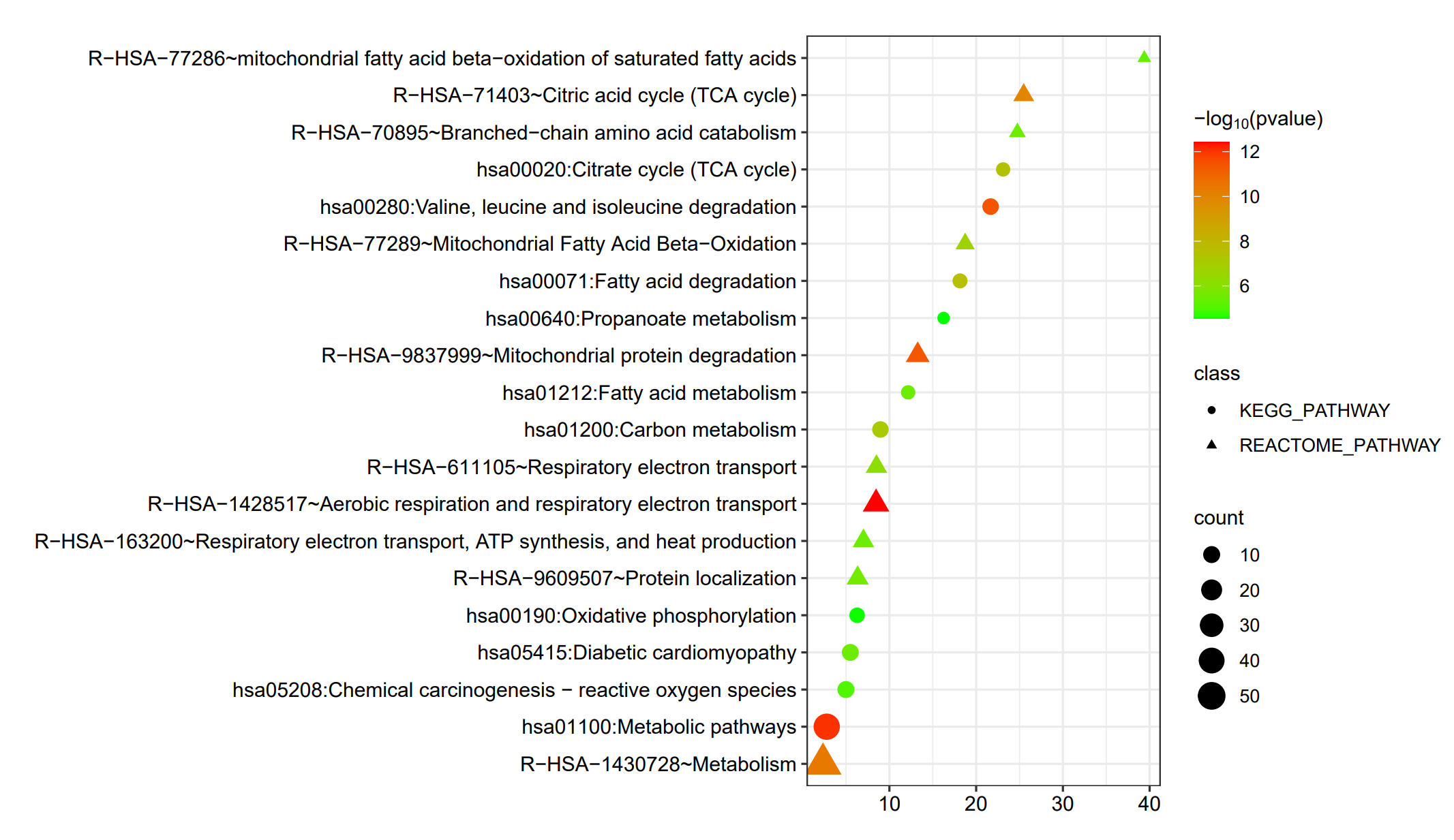


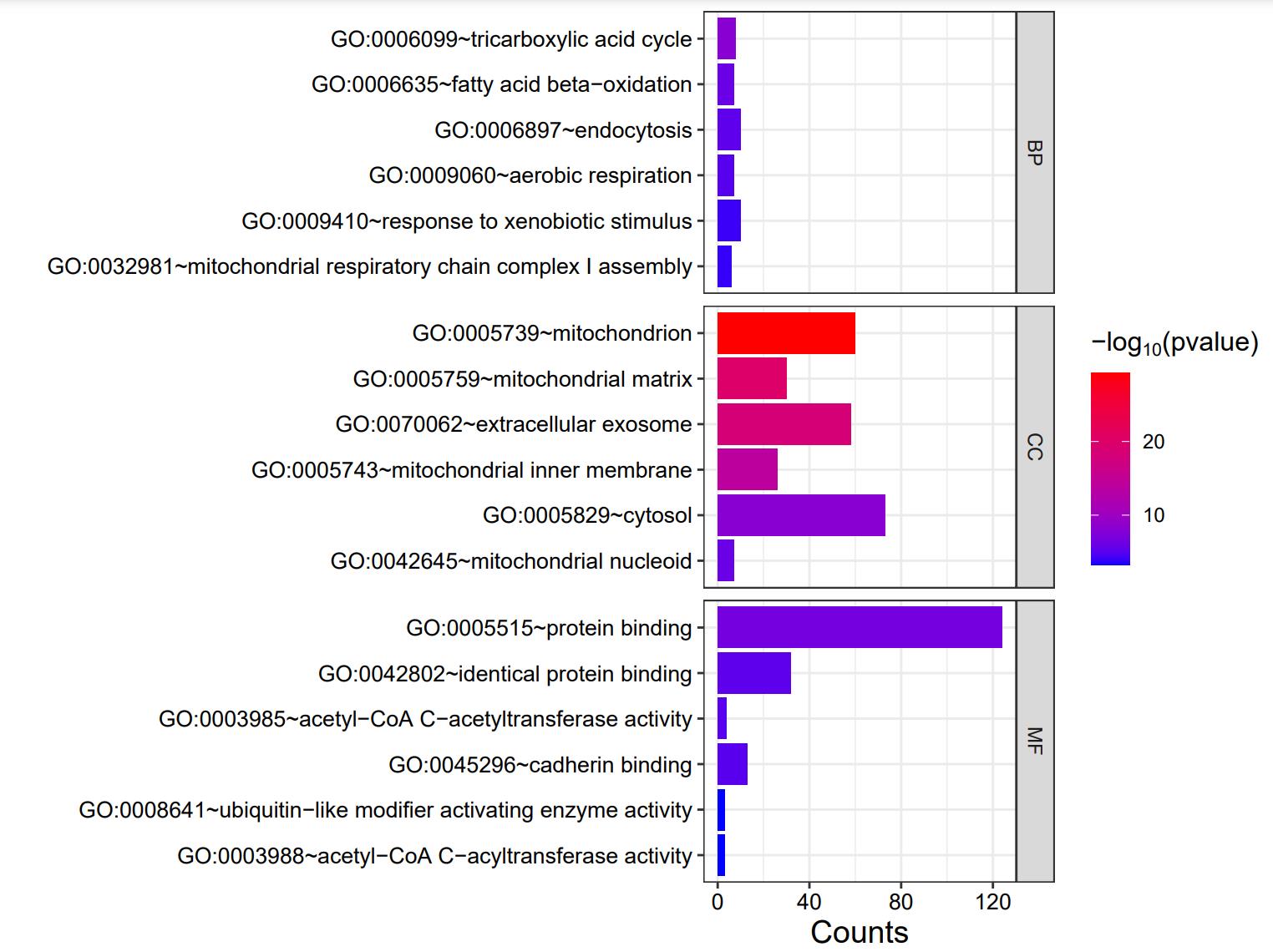


**Supplementary Fig.1.** **Proteomic KEGG and GO results of PSTK overexpression in HK-2 cells**


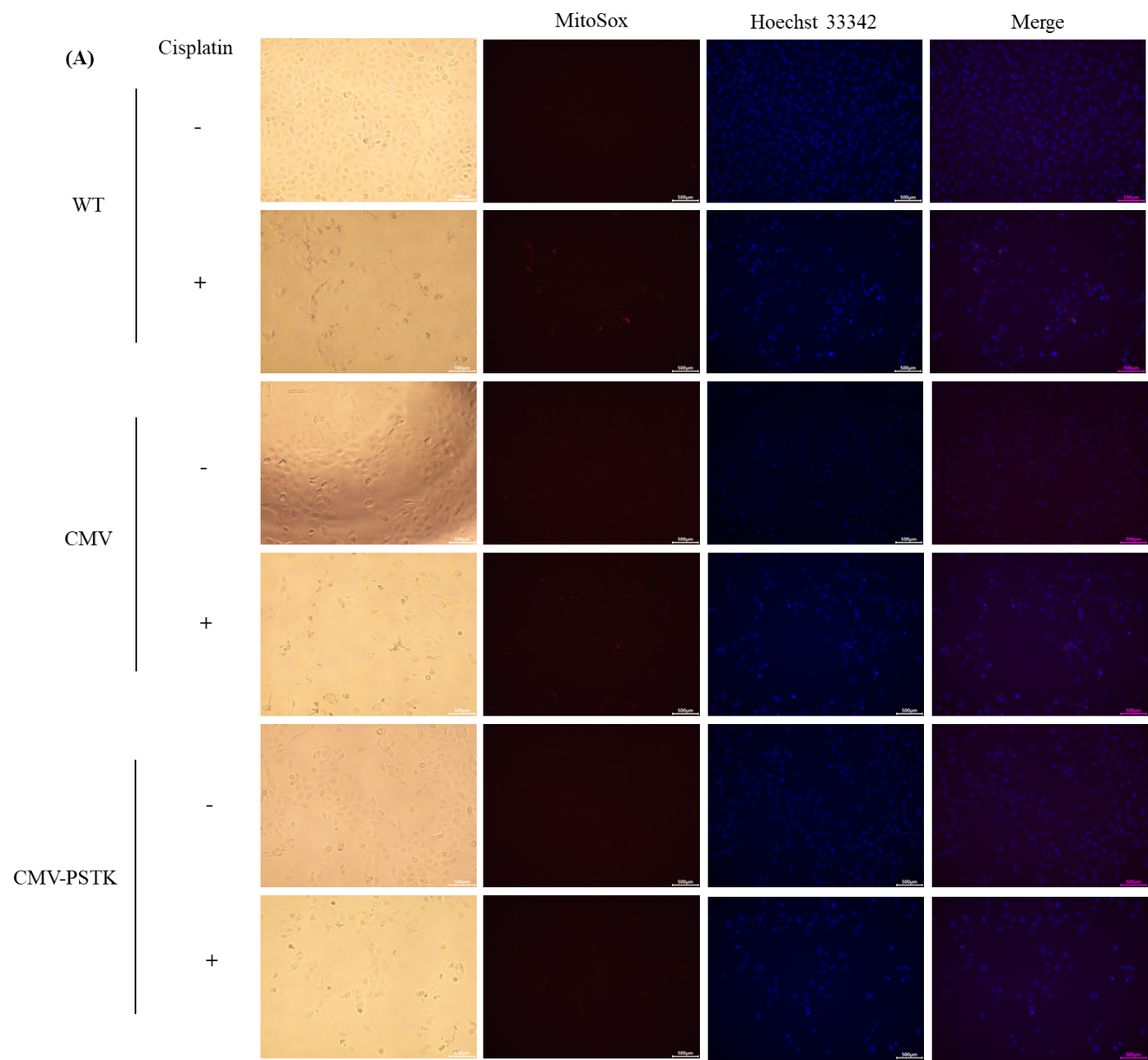


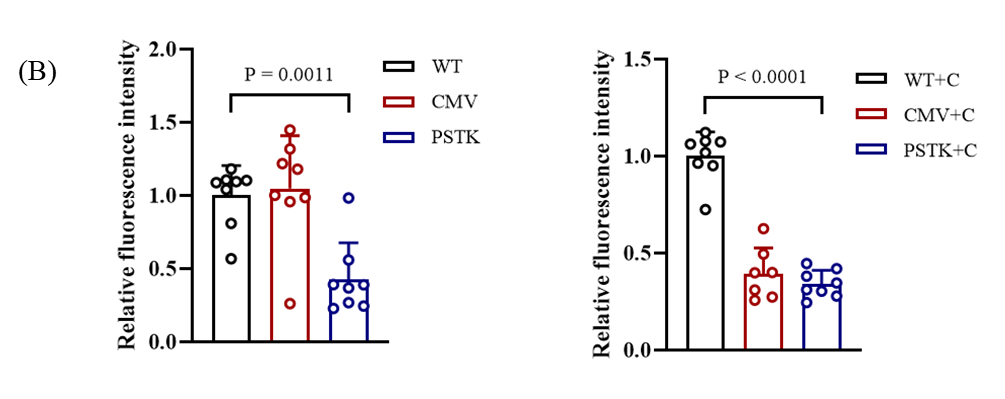


**Supplementary Fig.2. PSTK can reduce ROS in mitochondria**

(**A**)Represent fluorescence images of MitoSox staining in HK-2 cells. Hoechst 33342 (Blue) and MitoSox (Red). Scale bars represent 500 μm. (**B**) Quantifcation of mitochondrial ROS levels using MitoSox-Red fluorescent probes. The ROS level was measured by average cell fluorescence intensity by fluorescence microscopy(*n*=8). All the data were repeated 3 times and represented as the mean±SEM, P-value was determined by one-way ANOVA with Dunnett’s post hoc correction.

**Measurement of mitochondrial ROS level**

The mitochondrial ROS levels were measured using a MitoSOX Red (MCE, HY-D1055) according to the manufacturer’s instructions. Different groups of HK-2 cells were seeded in 6-well plate, and after adhesion, cells were treated with 15μg/ml cisplatin for 24h. Then cells were incubated with 5 μM MitoSOX Red solution for 30 min in dark at 37℃ with 5% CO2 incubator. The cell nucleus was stained with Hoechst 33342 (C1025, Beyotime, China). The red fluorescence emitted by MitoSOX in HK-2 cells was detected with fluorescence microscope ().
